# Surgical Experience from the STASEY Study of Emicizumab Prophylaxis in People with Hemophilia A with Factor VIII Inhibitors

**DOI:** 10.1055/s-0043-1777766

**Published:** 2024-01-12

**Authors:** Giancarlo Castaman, Flora Peyvandi, Johanna A. Kremer Hovinga, Roger E.G. Schutgens, Susan Robson, Katya Moreno, Víctor Jiménez-Yuste

**Affiliations:** 1 Center for Bleeding Disorders and Coagulation, Careggi University Hospital, Florence, Italy; 2 IRCCS Fondazione Ca' Granda Ospedale Maggiore Policlinico, Angelo Bianchi Bonomi Hemophilia and Thrombosis Center, Milan, Italy; 3 Department of Pathophysiology and Transplantation, Università degli Studi di Milano, Milan, Italy; 4 Department of Hematology and Central Hematology Laboratory, Bern University Hospital, University of Bern, Bern, Switzerland; 5 Center for Benign Haematology, Thrombosis and Haemostasis Van Creveldkliniek, University Medical Center Utrecht, Utrecht University, Utrecht, Netherlands; 6 PD Data Science, F. Hoffmann-La Roche Ltd, Basel, Switzerland; 7 Global Product Development/Medical Affairs, F. Hoffmann-La Roche Ltd, Basel, Switzerland; 8 Department of Hematology, Hospital Universitario La Paz, Autónoma University, Madrid, Spain

**Keywords:** clinical trial, emicizumab, hemophilia A, hemostasis, surgeries

## Abstract

**Background** Guidelines surrounding emicizumab prophylaxis and perioperative treatment for people with hemophilia A (PwHA) with factor (F)VIII inhibitors undergoing surgeries are limited. The phase IIIb multicenter, single-arm STASEY study evaluated safety and tolerability of emicizumab prophylaxis in PwHA aged ≥12 years with FVIII inhibitors. This analysis assesses surgeries during study conduct, associated hemophilia medications, and postoperative bleeds (treated and untreated).

**Methods** PwHA with FVIII inhibitors received emicizumab 3.0 mg/kg/week for 4 weeks, then 1.5 mg/kg/week until 2 years. Surgeries were managed and documented by treating physicians. Bleeds and treatments were recorded by physicians and participants.

**Results** Forty-six participants had ≥1 on-study surgery, 37 underwent 56 minor surgeries, and 13 underwent 22 major surgeries. Four participants underwent both minor and major surgeries. Of 18 (81.8%) and 4 (18.2%) major surgeries managed with/without additional hemostatic medication, 33.3 and 25.0% were associated with a treated postoperative bleed, respectively. Of 24 (42.9%) and 32 (57.1%) minor surgeries managed with/without additional hemostatic medication, 15.6 and 25.0% were associated with a treated postoperative bleed, respectively. Recombinant activated FVII was the most common medication for prophylaxis and bleed treatment. There were no thrombotic microangiopathies (TMAs). One hypertrophic clot, considered unrelated to emicizumab, occurred following tooth extraction.

**Conclusion** In this challenging population with a high bleeding risk, major surgeries were performed in PwHA receiving emicizumab with/without additional hemostatic medication. Postoperative bleeds occurred following 59.1% of major surgeries; 53.8% were treated. No arterial/venous thrombotic events or TMAs occurred due to concomitant emicizumab and bypassing agents.

**Trial registration** This trial is registered at ClinicalTrials.gov (NCT03191799).

## Introduction

Hemophilia A (HA) is a congenital blood disorder caused by a deficiency or dysfunction of coagulation factor (F)VIII. 1 Historically, people with HA (PwHA) were treated with recombinant FVIII products or plasma-derived concentrates; however, up to 30% of people with severe HA develop FVIII inhibitors, rendering FVIII replacement therapy ineffective. 2 Treatment options for PwHA with FVIII inhibitors include activated prothrombin complex concentrate (aPCC) and recombinant activated FVII (rFVIIa), known as bypassing agents (BPAs). 2 3

Joint damage and progressive arthropathy are common among PwHA, 4 5 and may result in the need for surgical procedures. 6 Performing such procedures in PwHA requires special considerations due to the risk of prolonged bleeding, and also carries a greater risk of delayed wound healing and infection following surgery. 7 8 In order to control bleeding during surgery, FVIII is administered to PwHA without FVIII inhibitors, while PwHA with FVIII inhibitors typically receive BPAs and/or antifibrinolytic agents. 9 The hemostatic efficacy and safety of bolus and continuous infusions of rFVIIa have previously been investigated in the surgical management of people with hemophilia A or B with FVIII or FIX inhibitors; this randomized clinical trial highlighted the risks associated with surgeries in this population, notably serious adverse events (AEs) related to inadequate hemostasis in the postoperative period. 10 Hence, surgeries in PwHA with FVIII inhibitors remain challenging; surgeons are commonly reluctant to perform surgeries in PwHA with high-titer FVIII inhibitors (≥5 Bethesda units/mL) due to the high bleeding risk. 11

In 2018, the first non-factor prophylactic therapy for PwHA was approved 12 : the bispecific humanized monoclonal antibody emicizumab, which bridges activated FIX and FX to substitute for deficient activated FVIII. 13 Emicizumab has been approved for the treatment of PwHA with or without FVIII inhibitors, including people with all severities of HA by the U.S. Food and Drug Administration 12 and people with severe or moderate HA with a severe bleeding phenotype by the European Medicines Agency. 14 At the time of writing, guidelines surrounding emicizumab prophylaxis and perioperative treatment options for PwHA with FVIII inhibitors undergoing surgeries are limited. 9 Given the thrombotic events (TEs) and thrombotic microangiopathies (TMAs) that occurred in the HAVEN 1 trial subsequent to concomitant aPCC given at doses of >100 IU/kg/24 hours, recommended practice is to avoid concomitant administration of aPCC with emicizumab, if possible. If aPCC is required, it should be limited to 50 IU/kg as a first dose and a maximum of 100 IU/kg within a 24-hour period. 15 The Italian Association of Haemophilia Centres have advised that low doses of aPCC could be considered in the case of an unplanned surgery, to prevent bleeding in PwHA with FVIII inhibitors for whom rFVIIa has been ineffectual. 16 Other publications have outlined or included suggested guidance for surgical management of PwHA with FVIII inhibitors receiving emicizumab but recognized the scarcity of large datasets to provide sufficient evidence for official guidelines. 17 18 Data from a pooled analysis from the HAVEN 1 to 4 clinical trials, which included information collated from >200 surgeries in PwHA found that minor and major surgeries were safely performed in PwHA receiving emicizumab prophylaxis, irrespective of the presence of FVIII inhibitors. 19 Nevertheless, the authors acknowledged that further evidence is needed to guide the development of guidelines for surgeries in PwHA receiving emicizumab. 19

The phase IIIb STASEY study was conducted to evaluate the safety and tolerability of emicizumab prophylaxis in PwHA aged ≥12 years with FVIII inhibitors. 20 The STASEY study was not designed to capture surgical outcomes; however, a large proportion of participants had to undergo nonplanned surgical interventions due to the long duration of the study and the resulting dataset offers a wealth of experience, which we report here. The objective of this analysis is to assess the safety of emicizumab prophylaxis with and without additional hemostatic medication in a surgical context.

## Methods

### Study Design and Participants

The STASEY study design and population have been previously described. 20 In brief, this was a phase IIIb, multicenter, single-arm study (clinicaltrials.gov identifier NCT03191799). Enrolled PwHA with FVIII inhibitors were aged ≥12 years with congenital HA; they received 3.0 mg/kg/week of emicizumab for 4 weeks, then 1.5 mg/kg/week of emicizumab for the remainder of the 2-year treatment period.

This study was conducted in full accordance with the International Conference on Harmonization (ICH) E6 guideline for Good Clinical Practice and the principles of the Declaration of Helsinki. The study complied with the requirements of the ICH E2A guideline (Clinical Safety Data Management: Definitions and Standards for Expedited Reporting). The trial protocol was approved by an independent institutional review board/ethics committee at each site prior to study start. An independent Data Monitoring Committee was established to monitor safety and study conduct. All participants provided written informed consent prior to any study-related procedures being performed; PwHA with FVIII inhibitors aged <18 years had informed consent provided by their legal guardian.

### Data Collection

No protocol guidance was available for surgical management. Surgical procedures were managed by the treating physician. Procedures were classed as minor or major, as defined by Santagostino et al. 21 Minor surgeries were defined as an invasive procedure in which manipulation occurred only on skin, mucous membranes, or superficial connective tissues. 21 Major surgeries were defined as an invasive procedure whereby a body cavity was entered, a mesenchymal barrier was crossed, a fascial plane was opened, an organ removed, and/or normal anatomy was operatively altered. 21

The on-study surgery population included participants who received at least one dose of emicizumab and had at least one surgical procedure during the study. Participants undergoing surgery ≥28 days after their last dose of emicizumab were no longer considered to be “on study” and were thus omitted from the on-study surgery population.

The following data were collected and summarized for the on-study surgery population, for minor and major surgeries separately, and by surgery category (arthroplasty; central venous access device [CVAD] related; dental; joint; other): surgeries during study conduct; hemophilia medications related to these surgeries (as collected on the Related Hemophilia Medication Log as part of the electronic Case Report Form [eCRF] completed by the treating physician, or the Bleed and Medication Questionnaire [BMQ] completed weekly by the participant using a handheld device); and occurrence of postoperative bleeds (treated and untreated), that is, bleeds related to a surgery or procedure (collected on the eCRF and/or BMQ). Data on tranexamic acid were collected under “Concomitant Medication.”

Medications recorded encompassed rFVIIa, aPCC, standard half-life and extended half-life FVIII, fresh frozen plasma/whole blood, and cryoprecipitate. The cumulative dose and number of infusions of additional hemostatic medications given prophylactically or to treat a postoperative bleed per surgery were summarized for each hemophilia medication separately. The duration of treatment (in days) was summarized for additional prophylactic hemostatic medication received.

A standardized definition of a bleed, adapted from the criteria defined by the FVIII and FIX Subcommittee of the Scientific and Standardization Committee of the International Society on Thrombosis and Haemostasis was used. 22 Treated bleeds were defined as bleeds followed by a hemophilia medication reported to be a “treatment for bleed,” irrespective of the time between the treatment and the preceding bleed.

### Study Sponsorship

The STASEY study was designed by the sponsor, F. Hoffmann-La Roche Ltd, and data were collected by the participants and the treating physicians. Data analysis was conducted by the study statistician and clinical pharmacologist (both employed by the sponsor), who vouch for the completeness and accuracy of the data and analysis. Specific direction from the authors informed the development of the first draft of the manuscript by Ashfield MedComms (funded by F. Hoffmann-La Roche Ltd.) and that draft was subsequently critically reviewed and revised by the authors.

## Results

### Study Population

**Fig. 1:**
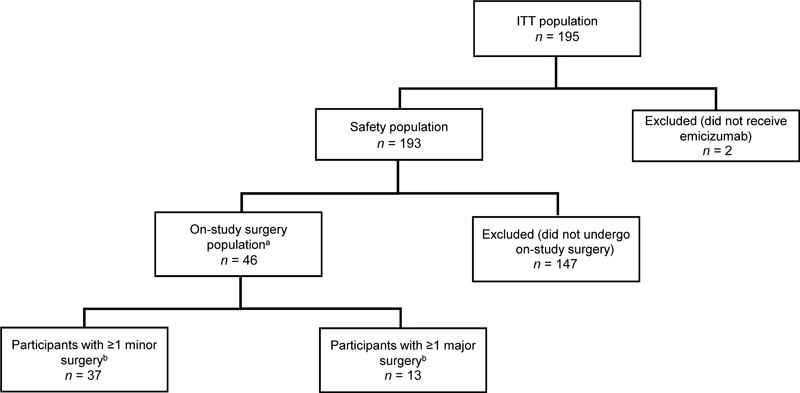
Participant disposition for the STASEY surgeries analysis. ^a^ In the safety population, one participant underwent a surgical procedure, but this occurred 91 days after their last dose of emicizumab and so the participant was excluded from the on-study surgery population. ^b^ Participants with both minor and major surgeries are counted in both categories. ITT population, intent-to-treat population.

At data cutoff (January 13, 2021), 46 of the 193 participants who received emicizumab had undergone a total of 78 on-study surgeries ( Fig. 1 ). Thirty-seven participants underwent 56 minor surgeries, and 13 had 22 major surgeries. This includes four participants who underwent both minor and major surgeries. Within this population, the median (interquartile range [IQR]) age was 31.5 (20.0–51.0) years, and the majority were aged ≥18 to <65 years ( *n* = 36, 78.3%; Table 1 ). Most participants had severe HA ( *n* = 39, 84.8%), five had moderate HA (10.9%), and two had mild HA (4.3%). All participants had current and neutralizing FVIII inhibitors.

**Table 1:** Baseline demographics and characteristics

	1.5 mg/kg emicizumab QW ( *n* = 46)
**Age (years),** ***n***	
Mean (SD)	36.5 (18.7)
Median (IQR)	31.5 (20.0–51.0)
**Age group (years),** ***n*** **(%)**	
≥12 to <18	7 (15.2)
≥18 to <65	36 (78.3)
≥65	3 (6.5)
**Gender,** ***n*** **(%)**	
Male	46 (100)
**Race,** ***n*** **(%)**	
Asian	5 (10.9)
Black or African American	2 (4.3)
White	37 (80.4)
Unknown	2 (4.3)
**Ethnicity,** ***n*** **(%)**	
Hispanic or Latino	1 (2.2)
Not Hispanic or Latino	41 (89.1)
Not reported	2 (4.3)
Unknown	2 (4.3)
**Hemophilia severity at baseline,** ***n*** **(%)**	
Mild	2 (4.3)
Moderate	5 (10.9)
Severe	39 (84.8)
**Prior hemophilia treatment in last 24 weeks,** ***n*** ** (%) a**	
aPCC	25 (54.3)
rFVIIa	22 (47.8)
FVIII	6 (13.0)
Other	1 (2.2) b
**Highest historical inhibitor titer,** ***n*** **(%)**	
Median (range)	168 (11000.0)
<5 BU/mL	2 (4.3)
≥5 BU/mL	44 (95.7)
**Previously treated with ITI,** ***n*** **(%)**	
Yes	24 (52.2)
No	22 (47.8)

Abbreviations: aPCC, activated prothrombin complex concentrate; BU, Bethesda units; FVIII, factor VIII; ITI, immune tolerance induction; IQR, interquartile range; QW, once weekly; rFVIIa, activated recombinant FVII; SD, standard deviation.

a Multiple answers are possible.

b Tranexamic acid.

Participants started with a loading dose of 3 mg/kg/week of emicizumab for 4 weeks.

### All Minor Surgeries

In total, 56 minor surgeries were performed in 37 participants ( Table 2 ). Overall median (IQR) emicizumab exposure time prior to minor surgery was 352.5 (198.5–513.0) days. One participant underwent minor surgery (suture insertion and skin laceration) on day 9, prior to completion of the loading phase.

**Table 2:** Minor surgeries within the on-study surgery population

	Minor surgeries				
	CVAD d	Dental e	Joint f	Other g	All minor
**Participants with at least one surgery,** ***n***	8	14	3	17	37
**Total surgeries,** ***n***	9	20	4	23	56
**Surgeries managed without additional prophylactic hemostatic medication,** ***n*** ** (%) ab**	**5 (55.6)**	**13 (65.0)**	**2 (50.0)**	**12 (52.2)**	**32 (57.1)**
No postoperative bleeds, *n* (%) c	1 (20.0)	6 (46.2)	2 (100.0)	8 (66.7)	17 (53.1)
Postoperative bleeds, *n* (%) c	4 (80.0)	7 (53.8)	0 (0.0)	4 (33.3)	15 (46.9)
Treated postoperative bleeds, *n* (%) c	2 (40.0)	2 (15.4)	0 (0.0)	1 (8.3)	5 (15.6)
**Surgeries managed with additional prophylactic hemostatic medication (rFVIIa, FVIII, and/or aPCC),** ***n*** ** (%) ab**	**4 (44.4)**	**7 (35.0)**	**2 (50.0)**	**11 (47.8)**	**24 (42.9)**
No postoperative bleeds, *n* (%) c	1 (25.0)	3 (42.9)	2 (100.0)	7 (63.6)	13 (54.1)
Postoperative bleeds, *n* (%) c	3 (75.0)	4 (57.1)	0 (0.0)	4 (36.4)	11 (45.8)
Treated postoperative bleeds, *n* (%) c	1 (25.0)	3 (42.9)	0 (0.0)	2 (18.2)	6 (25.0)

Abbreviations: aPCC, activated prothrombin complex concentrate; BMQ, Bleed and Medication Questionnaire; CVAD, central venous access device; FVIII, factor VIII; rFVIIa, activated recombinant FVII.

Data collected from the BMQ completed by participants.

a Percentages are calculated out of the total number of that type of surgery (e.g., CVAD, dental, joint, other, all).

b Tranexamic acid was also given for 18 procedures (4 dental and 2 other procedures where it was used in addition to other prophylactic hemostatic medications; and 2 CVAD, 8 dental, and 2 other procedures where it was used without any other prophylactic hemostatic medication).

c Percentages are calculated out of the number of that type of surgery (e.g., CVAD, dental, joint, other, all) managed with or without additional prophylactic hemostatic medication.

d Central venous catheter removal ( *n* = 5); abscess drainage ( *n* = 2); central venous catheterization ( *n* = 1); catheterization venous ( *n* = 1).

e Tooth extraction ( *n* = 16, one with dental implantation and one with orthodontic procedure); suture insertion ( *n* = 1); thrombectomy ( *n* = 1); tooth avulsion ( *n* = 1); tooth repair ( *n* = 1).

f Aspiration joint ( *n* = 1); brachytherapy ( *n* = 1); joint fluid drainage ( *n* = 1); synoviorthesis ( *n* = 1).

g Debridement ( *n* = 3); hematoma evacuation ( *n* = 2); sebaceous cyst excision ( *n* = 2); suture insertion ( *n* = 2); skin wound ( *n* = 2); arteriovenous fistula operation ( *n* = 1); chemocauterization ( *n* = 1); cyst removal ( *n* = 1); fistula repair ( *n* = 1); laser eye surgery ( *n* = 1); inguinal hernia repair ( *n* = 1); papilloma excision ( *n* = 1); tumor excision ( *n* = 1); skin graft ( *n* = 1); skin graft, wound closure, and wound treatment ( *n* = 1); skin lesion removal ( *n* = 1); wound closure and wound treatment ( *n* = 1).

Twenty-four (42.9%) minor surgeries were managed with additional prophylactic hemostatic medication (rFVIIa, FVIII, and/or aPCC). Of these, 22 (91.7%) were managed with prophylactic rFVIIa ( Table 3 ), including 4 CVAD surgeries, 6 dental, 2 joint, and 10 other surgeries. The median (IQR) number of rFVIIa infusions given for minor surgeries was 2.0 (1.0–9.0), with a median (IQR) cumulative dose of 147.2 (90.9–465.8) μg/kg, over a median (IQR) of 1.0 (1.0–5.0) days. The other two surgeries (one dental, one other) were managed using standard half-life FVIII concentrate. Out of the 24 minor surgeries managed with rFVIIa or standard half-life FVIII concentrate, tranexamic acid was also given in 6 surgeries. Thirty-two of the 56 minor surgeries (57.1%) were managed without additional prophylactic hemostatic medication. Tranexamic acid was given in 12 of these surgeries.

**Table 3:** Type and dose of additional prophylactic coagulation factor administered for minor surgeries

	CVAD ( *n* = 9)	Dental ( *n* = 20)	Joint ( *n* = 4)	Other ( *n* = 23)	Total ( *n* = 56)
**rFVIIa**					
Surgeries associated with additional prophylactic treatment, *n*	**4**	**6**	**2**	**10**	**22**
Number of doses					
Mean (SD)	3.0 (4.0)	3.2 (4.4)	13.5 (16.3)	10.4 (17.6)	7.4 (13.0)
Median (IQR)	1.0 (1.0–5.0)	1.0 (1.0–3.0)	13.5 (2.0–25.0)	2.5 (1.0–16.0)	2.0 (1.0–9.0)
Min–Max	1.0–9.0	1.0–12.0	2.0–25.0	1.0–57.0	1.0–57.0
Total cumulative dose					
Mean (SD)	171.1 (208.3)	305.6 (359.7)	858.0 (1,038.8)	914.6 (1,636.4)	608.2 (1,161.6)
Median (IQR)	97.4 (23.9–318.4)	182.8 (90.0–273.7)	858.0 (123.5–1,592.6)	192.4 (91.6–1,327.4)	147.2 (90.9–465.8)
Min–Max	23.9–465.8	87.5–1,016.9	123.5–1,592.6	66.5–5,320.0	23.9–5,320.0
Duration (days)					
Mean (SD)	2.0 (2.0)	1.7 (1.6)	3.0 (2.8)	6.0 (8.1)	3.8 (5.8)
Median (IQR)	1.0 (1.0–3.0)	1.0 (1.0–1.0)	3.0 (1.0–5.0)	1.0 (1.0–14.0)	1.0 (1.0–5.0)
Min–Max	1.0–5.0	1.0–5.0	1.0–5.0	1.0–22.0	1.0–22.0
**aPCC**					
Surgeries associated with additional prophylactic treatment, *n*	**0**	**0**	**0**	**0**	**0**
**Standard half-life FVIII concentrate**					
Surgeries associated with additional prophylactic treatment, *n*	**0**	**1**	**0**	**1**	**2**
Number of doses					
Mean (SD)	–	1.0 (NE)	–	2.0 (NE)	1.5 (0.7)
Median (IQR)	N/A	1.0 (1.0–1.0)	N/A	2.0 (2.0–2.0)	1.5 (1.0–2.0)
Min–Max	–	1.0–1.0	–	2.0–2.0	1.0–2.0
Total cumulative dose					
Mean (SD)	–	62.9 (NE)	–	313.3 (NE)	188.1 (177.1)
Median (IQR)	N/A	62.9 (62.9–62.9)	N/A	313.3 (313.3–313.3)	188.1 (62.9–313.3)
Min–Max	–	62.9–62.9	–	313.3–313.3	62.9–313.3
Duration (days)					
Mean (SD)	–	1.0 (NE)	–	12.0 (NE)	6.5 (7.8)
Median (IQR)	N/A	1.0 (1.0–1.0)	N/A	12.0 (12.0–12.0)	6.5 (1.0–12.0)
Min–Max	–	1.0–1.0	–	12.0–12.0	1.0–12.0

Abbreviations: aPCC, activated prothrombin complex concentrate; BMQ, Bleed and Medication Questionnaire; CVAD, central venous access device; IQR, interquartile range; FVIII, factor VIII; N/A, nonapplicable; NE, not evaluated; rFVIIa, recombinant activated FVII; SD, standard deviation.

Data collected from the BMQ completed by participants.

For some participants, the detail of all infusions was not collected in the BMQ (several infusions grouped into one entry), leading to a number of doses lower than the reality.

Postoperative bleeds were reported in 26/56 (46.4%) minor surgeries: 11/24 (45.8%) and 15/32 (46.9%) of the surgical procedures managed with and without additional prophylactic hemostatic medication, respectively. In 11/26 (42.3%) surgeries, these postoperative bleeds were treated (6/11 and 5/15 associated with surgeries managed with and without additional prophylactic hemostatic medication, respectively). Nine out of 11 (81.8%) were treated with rFVIIa, 1 (9.1%) was treated with aPCC, and 1 (9.1%) with standard half-life FVIII concentrate ( Table 4 ). The median (IQR) number of rFVIIa infusions given as treatment for postoperative bleeds associated with minor surgeries was 1.0 (1.0–2.0).

**Table 4:** Type and dose of treatment administered for postoperative bleeds associated with minor surgeries

	CVAD ( *n* = 9)	Dental ( *n* = 20)	Joint ( *n* = 4)	Other ( *n* = 23)	Total ( *n* = 56)
**rFVIIa**					
Surgeries associated with treatment for postoperative bleeds, *n*	**3**	**3**	**0**	**3**	**9**
Number of doses					
Mean (SD)	2.0 (1.0)	1.0 (0.0)	–	3.7 (3.8)	2.2 (2.3)
Median (IQR)	2.0 (1.0–3.0)	1.0 (1.0–1.0)	N/A	2.0 (1.0–8.0)	1.0 (1.0–2.0)
Min–Max	1.0–3.0	1.0–1.0	–	1.0–8.0	1.0–8.0
Total cumulative dose					
Mean (SD)	526.4 (728.6)	61.8 (40.2)	–	1,189.4 (1,826.5)	592.5 (1,099.1)
Median (IQR)	123.3 (88.4–1,367.5)	82.4 (15.4–87.5)	N/A	182.9 (87.5–3,297.7)	88.4 (87.5–182.9)
Min–Max	88.4–1367.5	15.4–87.5	–	87.5–3,297.7	15.4–3,297.7
**aPCC**					
Surgeries associated with treatment for postoperative bleeds, *n*	**0**	**1**	**0**	**0**	**1**
Number of doses					
Mean (SD)	–	1.0 (NE)	–	–	1.0 (NE)
Median (IQR)	N/A	1.0 (1.0–1.0)	N/A	N/A	1.0 (1.0–1.0)
Min–Max	–	1.0–1.0	–	–	1.0–1.0
Total cumulative dose					
Mean (SD)	–	109.5 (NE)	–	–	109.5 (NE)
Median (IQR)	N/A	109.5 (109.5–109.5)	N/A	N/A	109.5 (109.5–109.5)
Min–Max	–	109.5–109.5	–	–	109.5–109.5
**Standard half-life FVIII concentrate**					
Surgeries associated with treatment for postoperative bleeds, *n*	**0**	**1**	**0**	**0**	**1**
Number of doses					
Mean (SD)	–	1.0 (NE)	–	–	1.0 (NE)
Median (IQR)	N/A	1.0 (1.0–1.0)	N/A	N/A	1.0 (1.0–1.0)
Min–Max	–	1.0–1.0	–	–	1.0–1.0
Total cumulative dose					
Mean (SD)	–	62.9 (NE)	–	–	62.9 (NE)
Median (IQR)	N/A	62.9 (62.9–62.9)	N/A	N/A	62.9 (62.9–62.9)
Min–Max	–	62.9–62.9	–	–	62.9–62.9

Abbreviations: aPCC, activated prothrombin complex concentrate; BMQ, Bleed and Medication Questionnaire; CVAD, central venous access device; IQR, interquartile range; FVIII, factor VIII; N/A, nonapplicable; NE, not evaluated; rFVIIa, recombinant activated FVII; SD, standard deviation.

Data collected from the BMQ completed by participants.

For some participants, the detail of all infusions was not collected in the BMQ (several infusions grouped into one entry), leading to a number of doses lower than the reality.

Of the 38 minor surgeries for which hemostatic response grading was known, 25 (65.8%) were rated excellent, 12 (31.6%) were rated good/fair, and 1 (2.6%) was rated poor ( Supplementary Table S1 ).

### Minor Central Venous Access Device Surgeries

In total, there were nine CVAD surgeries, all minor ( Table 2 ). The most common surgery was CVAD removal ( *n* = 5). There were also two abscess drainage procedures. Four of the nine CVAD surgeries (44.4%) were managed with additional prophylactic hemostatic medication (all received rFVIIa; Table 3 ).

In total, 7/9 (77.7%) CVAD surgeries resulted in a postoperative bleed (3/4 of those managed with additional prophylactic hemostatic medication and 4/5 of those managed without additional prophylactic hemostatic medication). Three of these bleeds were treated (1/3 and 2/4 of those associated with surgeries managed with and without additional prophylactic hemostatic medication, respectively), all with rFVIIa ( Table 4 ).

### Minor Dental Surgeries

In total, there were 20 dental surgeries, all minor ( Table 2 ). The most common dental surgery was tooth extraction ( *n* = 16). Seven of the 20 dental surgeries (35.0%) were managed with additional prophylactic hemostatic medication (6 with rFVIIa and 1 with standard half-life FVIII concentrate; Table 3 ).

In total, 11/20 (55.0%) dental surgeries resulted in postoperative bleeds (4/7 of those managed with additional prophylactic hemostatic medication and 7/13 of those managed without). Five of these 11 bleeds were treated (3/4 and 2/7 resulting from surgeries managed with and without additional prophylactic hemostatic medication, respectively), 3 with rFVIIa, 1 with aPCC, and 1 with standard half-life FVIII concentrate ( Table 4 ).

### Minor Joint Surgeries

In total, there were four minor joint surgeries ( Table 2 ), including three joint punctures and one synoviorthesis. Two (50.0%) surgeries were managed with additional prophylactic hemostatic medication (both with rFVIIa; Table 3 ).

No minor joint surgery resulted in a postoperative bleed. One procedure, a joint fluid drainage, was managed with thromboprophylaxis given on the day of the surgery ( Supplementary Table S2 ).

### Other Minor Surgeries

In total, there were 23 other minor surgeries ( Table 2 ), of which 11 (47.8%) were managed with additional prophylactic hemostatic medication (10 with rFVIIa and 1 with standard half-life FVIII concentrate; Table 3 ).

Eight out of 23 surgeries (34.8%) resulted in postoperative bleeds (4 each for those managed with and without additional prophylactic hemostatic medication). Three of these were treated (2/4 and 1/4 of those associated with surgeries managed with and without additional prophylactic hemostatic medication, respectively), all with rFVIIa ( Table 4 ).

One inguinal hernia repair was managed with thromboprophylaxis given the day after the surgery ( Supplementary Table S2 ), and a hematoma evacuation was associated with a blood transfusion given on the same day as the surgery ( Supplementary Table S3 ).

### All Major Surgeries

A total of 22 major surgeries were reported in 13 participants ( Table 5 ). Median (IQR) emicizumab exposure time prior to major surgery was 527.0 (274.0–662.0) days.

**Table 5:** Major surgeries within the on-study surgery population

	Major surgeries		
	Arthroplasty d	Other e	All major
**Number of participants with at least one surgery,** ***n***	10	4	13
**Number of surgeries,** ***n***	13	9	22
**Surgeries managed without additional prophylactic hemostatic medication,** ***n*** ** (%) a**	**0 (0.0)**	**4 (44.4)**	**4 (18.2)**
No postoperative bleeds, *n* (%) b	N/A	3 (75.0)	3 (75.0)
Postoperative bleeds, *n* (%) b	N/A	1 (25.0)	1 (25.0)
Treated postoperative bleeds, *n* (%) b	N/A	1 (25.0)	1 (25.0)
**Surgeries managed with additional prophylactic hemostatic medication,** ***n*** ** (%) ac**	**13 (100.0)**	**5 (55.6)**	**18 (81.8)**
No postoperative bleeds, *n* (%) b	3 (23.1)	3 (33.3)	6 (33.3)
Postoperative bleeds, *n* (%) b	10 (76.9)	2 (22.2)	12 (66.7)
Treated postoperative bleeds, *n* (%) b	6 (46.2)	0 (0.0)	6 (33.3)

Abbreviation: BMQ, Bleed and Medication Questionnaire.

Data collected from the BMQ completed by participants.

a Percentages are calculated out of the total number of that type of surgery (e.g., arthroplasty, other, all).

b Percentages are calculated out of the number of that type of surgery (e.g., arthroplasty, other, all) managed with or without additional prophylactic hemostatic medication.

c Tranexamic acid was also given for five of these procedures (a hip arthroplasty in one participant; and two laparotomy procedures, a sigmoidectomy and a colostomy in another participant).

d Joint prosthesis ( *n* = 2), fracture treatment ( *n* = 2), arthrodesis ( *n* = 2, one with skin graft), hip surgery ( *n* = 1), leg amputation ( *n* = 1), bone operation ( *n* = 1), hip arthroplasty ( *n* = 1), knee arthroplasty ( *n* = 1), osteotomy and joint debridement ( *n* = 1), open reduction of fracture ( *n* = 1).

e Hemorrhoid operation ( *n* = 4), coronarography ( *n* = 1), sigmoidectomy ( *n* = 1), colostomy ( *n* = 1), laparotomy ( *n* = 1), polypectomy ( *n* = 1).

Most major surgeries ( *n* = 18, 81.8%) were managed with prophylactic hemostatic medications. Of these, 15 (83.3%) were managed with prophylactic rFVIIa, 2 (11.1%) were managed with aPCC, and 3 (16.7%) were managed with standard half-life FVIII concentrate ( Table 6 ), including 1 that was managed with both aPCC and rFVIIa and 1 managed with both standard half-life FVIII concentrate and rFVIIa. Tranexamic acid was also given in five of these major surgeries. The median (IQR) number of rFVIIa infusions was 20.0 (2.0–50.0), with a median (IQR) cumulative dose of 3253.1 (248.5–5508.5) μg/kg, given over a median (IQR) of 11.0 (2.0–22.0) days. The median (IQR) number of aPCC doses was 2.5 (2.0–3.0), with a median (IQR) cumulative dose of 65.9 (50.9–80.9) units/kg, given over a median (IQR) of 2.0 (1.0–3.0) days.

**Table 6:** Type and dose of additional prophylactic coagulation factor administered for major surgeries

	Arthroplasty ( *n* = 13)	Other ( *n* = 9)	Total ( *n* = 22)
**rFVIIa**			
Surgeries associated with additional prophylactic treatment, *n*	**11**	**4**	**15**
Number of doses			
Mean (SD)	42.2 (61.0)	23.0 (33.7)	37.1 (54.6)
Median (IQR)	30.0 (1.0–50.0)	8.5 (3.0–43.0)	20.0 (2.0–50.0)
Min–Max	1.0–214.0	2.0–73.0	1.0–214.0
Total cumulative dose			
Mean (SD)	2,446.9 (2,140.9)	8,673.7 (7,145.9)	4,107.4 (4,726.5)
Median (IQR)	2,625.0 (99.2–4,428.4)	7,101.7 (3,720.3–13,627.1)	3,253.1 (248.5–5,508.5)
Min–Max	80.0–5,945.2	1,932.2–18,559.3	80.0–18,559.3
Duration (days)			
Mean (SD)	20.7 (24.8)	7.8 (4.8)	17.3 (21.9)
Median (IQR)	11.0 (1.0–37.0)	8.0 (4.0–11.5)	11.0 (2.0–22.0)
Min–Max	1.0–78.0	2.0–13.0	1.0–78.0
**aPCC**			
Surgeries associated with additional prophylactic treatment, *n*	**1**	**1**	**2**
Number of doses			
Mean (SD)	3.0 (NE)	2.0 (NE)	2.5 (0.7)
Median (IQR)	3.0 (3.0–3.0)	2.0 (2.0–2.0)	2.5 (2.0–3.0)
Min–Max	3.0–3.0	2.0–2.0	2.0–3.0
Total cumulative dose			
Mean (SD)	80.9 (NE)	50.9 (NE)	65.9 (21.3)
Median (IQR)	80.9 (80.9–80.9)	50.9 (50.9–50.9)	65.9 (50.9–80.9)
Min–Max	80.9–80.9	50.9–50.9	50.9–80.9
Duration (days)			
Mean (SD)	3.0 (NE)	1.0 (NE)	2.0 (1.4)
Median (IQR)	3.0 (3.0–3.0)	1.0 (1.0–1.0)	2.0 (1.0–3.0)
Min–Max	3.0–3.0	1.0–1.0	1.0–3.0
**Standard half-life FVIII concentrate**			
Surgeries associated with additional prophylactic treatment, *n*	**2**	**1**	**3**
Number of doses			
Mean (SD)	2.0 (1.41)	1.0 (NE)	1.7 (1.2)
Median (IQR)	2.0 (1.0–3.0)	1.0 (1.0–1.0)	1.0 (1.0–3.0)
Min–Max	1.0–3.0	1.0–1.0	1.0–3.0
Total cumulative dose			
Mean (SD)	51.7 (71.4)	22.2 (NE)	41.9 (53.3)
Median (IQR)	51.7 (1.2–102.2)	22.2 (22.2–22.2)	22.2 (1.2–102.2)
Min–Max	1.2–102.2	22.2–22.2	1.2–102.2
Duration (days)			
Mean (SD)	1.5 (0.7)	1.0 (NE)	1.3 (0.6)
Median (IQR)	1.5 (1.0–2.0)	1.0 (1.0–1.0)	1.0 (1.0–2.0)
Min–Max	1.0–2.0	1.0–1.0	1.0–2.0

Abbreviations: aPCC, activated prothrombin complex concentrate; BMQ, Bleed and Medication Questionnaire; IQR, interquartile range; FVIII, factor VIII; NE, not evaluated; rFVIIa, recombinant activated FVII; SD, standard deviation.

Data collected from the BMQ completed by participants.

For some participants, the detail of all infusions was not collected in the BMQ (several infusions grouped into one entry), leading to a number of doses lower than the reality.

Thirteen of 22 major surgeries (59.1%) were associated with a postoperative bleed (12/18 and 1/4 of those managed with and without additional prophylactic hemostatic medication, respectively). However, only 7 of these postoperative bleeds (6/12 and 1/1 of those associated with surgeries managed with and without additional prophylactic hemostatic medication, respectively) were treated, all with rFVIIa. The median (IQR) number of rFVIIa infusions given to treat postoperative bleeds was 5.0 (1.0–51.0), with a median (IQR) cumulative dose of 473.0 (86.0–4009.6) μg/kg ( Table 7 ). One participant also received treatment with standard half-life FVIII concentrate.

**Table 7:** Type and dose of treatment administered for postoperative bleeds associated with major surgeries

	Arthroplasty ( *n* = 13)	Other ( *n* = 9)	Total ( *n* = 22)
**rFVIIa**			
Surgeries associated with treatment for postoperative bleeds, *n*	**6**	**1**	**7**
Number of doses			
Mean (SD)	29.7 (34.6)	5.0 (NE)	26.1 (33.0)
Median (IQR)	20.0 (1.0–51.0)	5.0 (5.0–5.0)	5.0 (1.0–51.0)
Min–Max	1.0–85.0	5.0–5.0	1.0–85.0
Total cumulative dose			
Mean (SD)	2,465.6 (2,887.1)	473.0 (NE)	2,180.9 (2,741.1)
Median (IQR)	1,787.0 (86.0–4,009.6)	473.0 (473.0–473.0)	473.0 (86.0–4,009.6)
Min–Max	40.0–7,083.9	473.0–473.0	40.0–7,083.9
**aPCC**			
Surgeries associated with treatment for postoperative bleeds, *n*	**0**	**0**	**0**
**Standard half-life FVIII concentrate**			
Surgeries associated with treatment for postoperative bleeds, *n*	**1**	**0**	**1**
Number of doses			
Mean (SD)	2.0 (NE)		2.0 (NE)
Median (IQR)	2.0 (2.0–2.0)	N/A	2.0 (2.0–2.0)
Min–Max	2.0–2.0		2.0–2.0
Total cumulative dose			
Mean (SD)	48.4 (NE)		48.4 (NE)
Median (IQR)	48.4 (48.4–48.4)	N/A	48.4 (48.4–48.4)
Min–Max	48.4–48.4		48.4–48.4

Abbreviations: aPCC, activated prothrombin complex concentrate; BMQ, Bleed and Medication Questionnaire; IQR, interquartile range; FVIII, factor VIII; N/A, nonapplicable; NE, not evaluated; rFVIIa, recombinant activated FVII; SD, standard deviation.

Data collected from the BMQ completed by participants.

For some participants, the detail of all infusions was not collected in the BMQ (several infusions grouped into one entry), leading to a number of doses lower than the reality.

Of the 13 major surgeries for which the hemostatic response grading was known, 6 (46.2%) were rated excellent, 3 (23.1%) were rated good, 3 (23.1%) were rated fair, and 1 (7.7%) was rated poor ( Supplementary Table S4 ).

### Major Arthroplasty Surgeries

In total, there were 13 arthroplasty surgeries ( Table 5 ), the most common of which were arthrodesis ( *n* = 2, 15.4%), fracture treatment ( *n* = 2, 15.4%), and joint prosthesis ( *n* = 2, 15.4%). All arthroplasties were managed with additional prophylactic hemostatic medication ( Table 6 ).

Ten arthroplasty surgeries (76.9%) resulted in a postoperative bleed, of which six (60.0%) were treated (all with rFVIIa, and one participant also received treatment with standard half-life FVIII concentrate; Table 7 ). One arthroplasty, a hip surgery, was managed with thromboprophylaxis given on the day of the surgery ( Supplementary Table S1 ). Four arthroplasties, including two fracture treatments, an open reduction of fracture (realignment of a broken bone during surgery), and an arthrodesis and skin graft, were associated with blood transfusions, given at varying times pre-, peri-, and/or postoperatively ( Supplementary Table S2 ).

### Other Major Surgeries

In total, there were nine other major surgeries ( Table 5 ). The most common other surgery was hemorrhoid operation ( *n* = 4, 44.4%). Five (55.6%) surgeries were managed with additional prophylactic hemostatic medication ( Table 6 ), and four (44.4%) were managed without.

Postoperative bleeds occurred in association with 3 surgeries (2/5 and 1/4 of those managed with and without additional prophylactic hemostatic medication, respectively). The postoperative bleed resulting from the surgery managed without additional prophylactic hemostatic medication was treated with rFVIIa ( Table 7 ).

One coronarography (following myocardial ischemia) was managed with thromboprophylaxis given on the same day as the surgery ( Supplementary Table S2 ).

### Adverse Events

No deaths occurred in the on-study surgery population. Thirteen AEs associated with emicizumab prophylaxis occurred in the on-study surgery population across the duration of the study. The majority of AEs were injection site reactions ( *n* = 7, 53.8%). Other AEs included catheter site abscess ( *n* = 1), contusion ( *n* = 1), postprocedural hematoma ( *n* = 1), fatigue ( *n* = 1), pruritus ( *n* = 1), and dysgeusia ( *n* = 1). The postprocedural hematoma and catheter site abscess were of grade 3 severity.

Although BPAs were used in conjunction with emicizumab for hemostatic cover of surgeries, there were no instances of TMA. A localized hypertrophic clot occurred at the site of a tooth extraction in one participant who was receiving a combination of antifibrinolytics in conjunction with rFVIIa, reported by the treating physician as a postoperative thrombosis. The event resolved after 6 days of treatment (etoricoxib, amoxicillin, and clavulanic acid) and was considered by the investigator to be unrelated to emicizumab and caused by concomitant medications (rFVIIa and tranexamic acid).

## Discussion

A number of PwHA with FVIII inhibitors receiving emicizumab prophylaxis underwent minor or major surgery throughout the duration of the STASEY study. More than half of the minor surgeries did not involve additional prophylactic hemostatic medication (32/56, 57.1%); 5/32 (15.6%) of these led to a treated postoperative bleed. CVAD surgeries were most likely to result in a postoperative bleed: these occurred in 3/4 (75%) surgeries managed with additional prophylaxis (1/3 [33.3%] of which were treated) and 4/5 (80%) surgeries managed without additional prophylaxis (2/4 [50%] of which were treated). The most common minor surgery was tooth extraction ( *n* = 16, 28.6%). On average, the total number of additional prophylactic rFVIIa infusions per surgery was low (median of 2), as was the average number of rFVIIa infusions given to treat postoperative bleeds (median of 1).

For many of the minor surgeries that took place in the STASEY study, emicizumab prophylaxis was able to provide adequate hemostatic control (97.4% of all minor surgeries for which the hemostatic grading was known were reported as “excellent” or “good/fair”). It is unclear from the data whether the additional prophylactic hemostatic medication administered was beneficial for managing minor surgeries, as the proportion of surgeries associated with a postoperative bleed was similar regardless of whether additional prophylactic hemostatic medication had been given or not. However, it should be noted that this may be influenced by the investigators being more likely to administer additional prophylactic hemostatic medication if they thought the individual undergoing surgery was more likely to bleed; for example, if they had shown a higher propensity to bleed in the past compared with other PwHA with FVIII inhibitors, or if the planned surgery was more invasive. Treatment decisions for surgeries that took place during the STASEY study were taken at the investigator's discretion, as no guidance was provided. Thus, the results of this study are likely to reflect real-world practice and the different perceptions of the investigators in managing surgeries in PwHA receiving emicizumab.

Importantly, no TMAs occurred, and emicizumab prophylaxis was not considered a causative factor in the TE that occurred in a participant undergoing a minor dental surgery. This was a hypertrophic clot following tooth extraction, which the investigator believed was related to concomitant rFVIIa and tranexamic acid. This event was categorized as a postoperative TE; however, it is not considered to be a typical intravascular TE. The clot resolved after 6 days of treatment, and the participant continued receiving emicizumab. No AEs were reported as a result of concurrent use of additional prophylactic hemostatic medication (rFVIIa, standard half-life FVIII concentrate, and/or aPCC) and emicizumab.

Major surgeries were managed with additional prophylactic hemostatic medication more often than minor surgeries, following the judgement of the treating physicians. The majority of major surgeries (18/22, 81.8%) were managed with additional prophylactic hemostatic medications and, overall, 7/22 (31.8%) major surgeries were associated with a treated postoperative bleed. This number must be interpreted carefully, taking into account the individual case characteristics, the complexity of the procedures, the bleeding tendency of the interventions, and the inherent risk of performing surgeries in PwHA with FVIII inhibitors. Complicated major surgeries like hip surgery, leg amputation, and arthrodesis required multiple doses of rFVIIa. Despite this, hemostatic control was maintained in most cases, according to the hemostatic response grading for surgeries where this was known, and no TMAs or TEs were observed.

Although 50% of postoperative bleeds associated with major surgeries managed with additional prophylactic hemostatic medication were treated, 50% were untreated; this may indicate that the bleeds did not require treatment, as bleeding is expected following major surgery even in people without hemophilia. 23 At the time the STASEY study commenced (2017), little evidence was available on the use of emicizumab prophylaxis concomitantly with BPAs such as rFVIIa and aPCC given on demand for surgical procedures. Hence, undertreatment may have occurred due to concerns of thrombotic risk if emicizumab were to be combined with BPAs, particularly aPCC. The findings of the STASEY study do not support any additional thrombotic risk when using emicizumab alongside BPAs, provided clinical guidelines are followed, especially with regard to aPCC 15 ; this is in-line with other reports since the initiation of the STASEY study. 19 24 25 26

The data reported herein support the growing evidence reported from clinical trials that emicizumab prophylaxis can provide hemostatic coverage during minor and major surgeries, with appropriate concomitant prophylactic hemostatic medication when required. In the first prospective study of PwHA with/without FVIII inhibitors undergoing minor surgeries, 26 a total of 13 participants underwent minor surgeries, and postoperative bleeds were reported in 30.8% of surgeries. The HAVEN 1 to 4 pooled analysis reported 215 minor surgeries in 115 PwHA with/without FVIII inhibitors and 18 major surgeries in 18 PwHA with/without FVIII inhibitors. 19 In that analysis, >65% of minor surgeries were managed without additional prophylactic coagulation factor. In total, 39 minor surgeries resulted in a postoperative bleed (18.1%), of which 22 (56.4%) were treated, while most major surgeries were managed with additional prophylactic hemostatic medication (83.3%); of these, only 1 (6.7%) resulted in a treated postoperative bleed.

Real-world experience is also aligned with the current data and prior clinical trial reports. In a retrospective analysis of real-world experience of PwHA with/without FVIII inhibitors receiving emicizumab, 31 surgeries (29 minor, 2 major) were performed in 25 participants. 24 Minor surgeries were managed with emicizumab alone or with additional FVIII or rFVIIa and tranexamic acid. 24 Major bleeding occurred in one instance (circumcision treated with only tranexamic acid in addition to emicizumab). 24 One major surgery (hip replacement) was associated with additional postoperative FVIII; the other, an explorative laparotomy, was managed with additional rFVIIa. 24 An additional real-world study reported cases of 20 minor and 5 major surgeries in 22 PwHA with/without FVIII inhibitors. 25 Four of the minor surgeries (20%) and all major surgeries were performed with additional hemostatic medication. 25 No major bleeding episodes occurred, and no thrombotic complications were reported. 25 In an observational study, 28 minor and 2 major surgeries were reported in 22 PwHA with/without FVIII inhibitors. 27 Minor surgeries included 21 CVAD removals, and major surgeries included intracranial ventricular shunt revision and posterior spinal fusion. 27 Three PwHA received up to two doses of unplanned factor postoperatively to treat minor bleeding events. 27 No participant discontinued emicizumab therapy, and there were no TEs or deaths reported. 27

In summation, the results of the STASEY study are aligned with data from other clinical studies and from real-world settings, adding to the growing evidence base that could be used to develop guidelines for management of surgeries in PwHA with/without FVIII inhibitors receiving emicizumab.

## Limitations

The STASEY study was not designed to analyze surgical outcomes and did not include any surgical end points; some surgical data were reported retrospectively. Consequently, pre-, intra-, and postoperative use of additional prophylactic hemostatic medication was documented by both physicians, in the Related Hemophilia Medication Form, and by participants in the BMQ, which may have led to inconsistencies in reporting. For some participants, the detail of all infusions was not collected in the BMQ (several infusions were grouped into one entry), leading to a number of reported doses lower than the reality, and uncertainty in when exactly these doses were administered in relation to the surgeries (e.g., a day before or directly before). Furthermore, data on usage of antifibrinolytics such as tranexamic acid were collected as part of the “Concomitant Medication Form” rather than systematically in the “Related Hemophilia Medication Form,” and so are not included in the additional prophylactic hemostatic medications usage.

It is possible that treated bleeds following surgeries may have been underreported in the STASEY study, as the additional hemostatic medications reported here were categorized as “prophylaxis” or “treatment for bleed” in the BMQ that was completed weekly by participants. Some participants with untreated bleeds received or continued to receive hemostatic medication during or following surgeries that was categorized as prophylaxis rather than bleed treatment; therefore, the data are reported according to participants' interpretation of the medication received and should be understood as such.

Finally, as mentioned, the STASEY study commenced in 2017 when experience in performing surgeries in PwHA with FVIII inhibitors receiving emicizumab prophylaxis was minimal, as yet unpublished, and limited to clinical trials. Clinical practice may have changed in the following years as the evidence base has grown. 19 24 25 26 27 As a result, the approach to surgical management taken by physicians when the STASEY study began may not be reflective of current practices: physicians may now have more confidence in using additional prophylactic coverage alongside emicizumab.

## Conclusion

This analysis reports data from the largest number of major surgeries performed in PwHA with FVIII inhibitors receiving emicizumab prophylaxis documented to date. Due to the study design and the heterogeneous approach of the investigators to managing these surgeries, it is not possible to draw firm conclusions on the efficacy of emicizumab prophylaxis in a surgical setting. All PwHA should be monitored closely after surgery due to the risk of bleeding. In this study, no AEs, including arterial/venous TEs or TMA, were reported as a result of the combination of emicizumab prophylaxis and aPCC, standard half-life FVIII concentrate, or repeated doses of rFVIIa during the management of surgeries.

Clinical judgement should be utilized to determine and manage a treatment plan. This should be characterized prior to surgery. These data provide further evidence that will hopefully inform the development of formal guidelines for performing surgery in PwHA with or without FVIII inhibitors receiving emicizumab and/or other treatments.

## Notes

**What is Known about This Topic?**

- Surgeries in people with HA with FVIII inhibitors are challenging due to the high risk of bleeding and limited treatment options.
- Emicizumab is a bispecific humanized monoclonal antibody that is approved for the treatment of people with HA with or without FVIII inhibitors.
- Guidelines surrounding emicizumab prophylaxis and perioperative treatment for people with HA with FVIII inhibitors undergoing surgeries are limited.

**What does This Paper Add?**

- This analysis reports on surgeries performed during the phase IIIb study STASEY in people with HA with FVIII inhibitors aged ≥12 years receiving emicizumab.
- Fifty-six minor surgeries were performed; 32 (57%) of these were managed without additional hemostatic medication, and of these 32, 17 (53%) were associated with no postoperative bleeds.
- Twenty-two major surgeries were performed; 18 (82%) of these were managed with additional hemostatic medication, and of these 18, 6 (33.3%) were associated with no postoperative bleeds.
