# Supplementary material for: Surgical Experience from the STASEY Study of Emicizumab Prophylaxis in People with Hemophilia A with Factor VIII Inhibitors
Source: TH Open. 2024 Jan 12;8(1):e42–54. doi: 10.1055/s-0043-1777766 (PMC10786707; doi:10.1055/s-0043-1777766)
Supplement: Supplementary file 1 — Supplementary Material [file 10-1055-s-0043-1777766-s23070030.pdf]

Supplementary Table S1 Summary of grading for hemostatic response associated with minor on-study surgeries

	Minor surgeries				
	CVADs	Dental	Joint	Other	All minor
**Participants with at least one surgery, *n***	8	14	3	17	37
**Total surgeries, *n***	9	20	4	23	56
**Grading of hemostatic response associated with surgeries**					
Excellent, *n* (%)	2 (22.2)	10 (50.0)	2 (50.0)	11 (47.8)	25 (44.6)
Good/Fair, *n* (%)	3 (33.3)	7 (35.0)	1 (25.0)	1 (4.3)	12 (21.4)
Fair, *n* (%)	0 (0)	0 (0)	0 (0)	0 (0)	0 (0)
Poor, *n* (%)	1 (11.1)	0 (0)	0 (0)	0 (0)	1 (1.8)
Unknown, n (%)	2 (22.2)	3 (15.0)	1 (25.0)	11 (47.8)	17 (30.4)
Missing, n (%)	1 (11.1)	0 (0)	0 (0)	0 (0)	1 (1.8)

Abbreviation: CVAD, central venous access device.
Data collected from the Related Hemophilia Medication Log completed by the treating physicians.

Supplementary Table S2 Details of thromboprophylaxis associated with surgeries during the STASEY study

Subject ID	Surgery	Surgery type	Relative starting study day for surgery	Thromboprophylaxis	Relative starting study day for medication	Indication
Participant A	Hip replacement	Major arthroplasty	310	Enoxaparin	310	Other
Participant B	Coronarography (due to myocardial ischemia)	Major other	235	Clopidogrel<br>Heparin	235	Adverse event
Participant C	Joint fluid drainage	Minor joint	167	Dalteparin	167	Prophylaxis
Participant D	Inguinal hernia repair	Minor other	667	Enoxaparin	668	Adverse event

Data collected from the Concomitant Medication Form completed by the treating physicians.

Supplementary Table S3 Details of transfusion requirements during and postsurgery throughout the STASEY study

Subject	Surgery	Surgery type	Relative starting study day for surgery	Transfusion	Relative starting study day for medication	Indication
Participant E	Fracture treatment	Major arthroplasty	337	Blood cells packed human	338, 353	Adverse event
Participant F	Open reduction of fracture[a]	Major arthroplasty	117	Blood cells packed human	116, 117, 120, 125, 129	Adverse event
Participant G	Hematoma evacuation	Minor other	561	Red blood cells concentrated	561	Adverse event
Participant H	Arthrodesis, skin graft	Major arthroplasty	527	Red blood cells concentrated	527	Adverse event
Participant I	Fracture treatment	Major arthroplasty	48	Blood whole	48, 49, 50	Adverse event

Data collected from the Concomitant Medication Form completed by the treating physicians.
[a]Realignment of a broken bone during surgery.

 © 2024. The Author(s).

Supplementary Table S4 Summary of grading for hemostatic response associated with major on-study surgeries

	**Major surgeries**		
	**Arthroplasty**	**Other**	**All major**
**Number of participants with at least one surgery, *n***	10	4	13
**Number of surgeries, *n***	13	9	22
**Grading of hemostatic response associated with surgeries**			
Excellent, *n* (%)	4 (30.8)	2 (22.2)	6 (27.3)
Good/Fair, *n* (%)	3 (23.1)	0	3 (13.6)
Fair, *n* (%)	3 (23.1)	0	3 (13.6)
Poor, n (%)	0 (0)	1 (11.1)	1 (4.5)
Unknown, *n* (%)	3 (23.1)	6 (66.7)	9 (40.9)

Data collected from the Related Hemophilia Medication Log completed by the treating physicians.

 © 2024. The Author(s).
